# Temporal resolution in fluorescence imaging

**DOI:** 10.3389/fmolb.2014.00011

**Published:** 2014-09-09

**Authors:** Partha Pratim Mondal

**Affiliations:** Nanobioimaging Laboratory, Department of Instrumentation and Applied Physics, Indian Institute of ScienceBangalore, India

**Keywords:** bioimaging, medical physics, fluorescence, imaging, fluorescence microscopy

## Abstract

Temporal resolution is a key factor for imaging rapidly occurring events in biology. In this feature article, I investigate an approximate estimate for determining the temporal resolution limit. The condition that led to this limit is, the time taken by the ensemble (99.9%) of excited molecules to relax to ground state, assuming all the emitted photons are detected. In a simplistic three-level system, the temporal resolution is, ≈3τ_*p*_, where τ_*p*_ = (log_*e*_10)/(*k_f_* + *k_nr_*) and, *k_f_* and *k_nr_* are respectively the radiative and non-radiative emission rates. This further assumes the ideal condition that, the quantum efficiency of the detector is unity and there are no other loses. We discuss few state-of-art microscopy techniques that are capable of high temporal resolution. This includes techniques such as multifocal multiphoton microscopy (MMM), multifocal plane microscopy, multiple excitation spot optical microscopy (MESO), multiplane microscopy and multiple light-sheet microscopy (MLSM).

## 1. Introduction

Fluorescence microscopy has emerged as one of the key imaging modalities in the last decade. This is predominantly due to its application in diverse fields ranging from biology to applied physics. As far as biotechnological applications are concerned, fluorescence microscope provides a real image. Analysis of these 3D high resolution images forms the basis for accurate interpretation of biological processes and provides a lot of important features such as, cell volume, local concentration, local pH values in the cellular compartments, presence of infective agents, the status of cell cycle and topography. Microscopy has becomes the prerequisite for many biotechnological studies such as, microspectroscopy, differentiation of organisms and micromanipulation techniques. Over the last few years, there has been a radical change with the advent of super-resolution microscopy. Briefly, super-resolution refers to imaging specimens with a resolution better than the classical resolution limit. This limit is popularly known as Abbe's diffraction limit (Abbe, 1873). Some of the prominent techniques that are capable of super-resolution are, PLAM (Betzig et al., 2006), fPALM (Hess et al., 2006), STORM (Rust et al., 2006), GSDIM (Fölling et al., 2008), STED (Hell and Wichmann, 1994), 4pi (Hell et al., 1994) and SI (Gustafsson, 2005). Of-late the field of super-resolution has expanded with the integration of super-resolution with light-sheet microscopy (Zanacchi et al., 2011; Deschout et al., 2014). Most of these techniques are capable of spatial super-resolution, some at the expense of poor temporal resolution. Specifically, this is true for localization techniques (PALM, fPALM, STORM, GSDIM). On the other hand, fluorescence imaging techniques that are capable of high temporal resolution are, multiphoton multifocal microscopy (MMM), multifocal plane microscopy, multiple excitation spot optical (MESO) microscopy and multiple light-sheet microscopy (MLSM). In the next few sections, I will elaborate on these technique and its working principle. In Section 2, I derive a simple expression for determining the temporal resolution when imaging with a specific dye. Section 3 deals with the description of imaging systems that are capable of high temporal resolution. We conclude the article in Section 4.

## 2. Temporal resolution limit in fluorescence microscopy

Temporal resolution of an imaging system depends upon many factors including, detector efficiency (Quantum Yield) and complex molecular properties. We realize that, the simple and logical way to quantify the temporal resolution can be derived by determining the time required by all excited molecules for completing a single excitation-emission cycle. This is based on the fact that, the temporal resolution is ultimately limited by the recycle time of the fluorescent molecule between ground and excited state, provided there is no photobleaching. Consider a simplistic three level system for the fluorophore (*S*_0_, *S*_1_, *T*_1_). The following processes occur in a three-level system: Excitation (*S*_0_ → *S*_1_), Internal Conversion (*S*_1_ (ν_*n*_ → ν_0_)) and Emission (*S*_1_ → *S*_0_) occurs in the timescales, 10^−15^ s, 10^−11^ s, and 10^−9^ s respectively. To simplify the process and to get an approximate estimate of the temporal resolution, one can neglect the time required for excitation and internal conversion as compared to the time required for emission process. So, the dominant sub-process that consumes maximum time in an excitation-emission cycle is essentially the emission time, and hence the temporal resolution is limited by the time required for all the excited molecules to return to the ground state (*S*_0_) from the excited state (*S*_1_). In other words, the temporal resolution is decided by the ensemble of molecules that gets excited at the focal volume. Let us consider that the local density of fluorescent molecules at the focal volume be ρ*_N_* and the focal volume be *V_psf_*. So, the total number of excited molecules in the focal volume is simply, ρ*_N_ V_psf_*. During the excitation process, the molecules that get excited to *S*_1_ are: σ ρ*_N_ V_psf_*, where σ is the absorption cross-section. The limit of temporal resolution is essentially the time required for all the excited molecules (i.e., ≈ 99.9%) to return to the ground state. This further assumes the ideal condition that, all the emitted photons are detected. One can take into account many other factors including the detector efficiency for better approximation of temporal resolution limit.

In a simplistic three-level system (*S*_0_, *S*_1_, *T*_1_), we assume that, molecules are in excited state *S*_1_. There are two major processes that result in deexcitation i.e., fluorescence (with, emission rate, *k_f_*) and non-radiative relaxation (with a rate, *k_nr_*). The governing equation for determining the excited and triplet state population are given by,

(1) $$\{\begin{array}{l} \frac{\partial }{\partial t}N_{S1}(t)=f_{ill}A\;N_{S0}(t)-(k_{f}+k_{nr})\;N_{S1}(t)\\ \frac{\partial }{\partial t}N_{T1}(t)=k_{nr}N_{S1}(t) \end{array}$$

with the additional constraint that, *N*_*S*0_ +*N*_*S*1_ + *N*_*T*1_ = 1. *f_ill_* ∝ *h*ν*_ill_k*_1_ is the photon flux of the excitation laser. *k_f_* + *k_nr_* is total emission rate (including both radiative and non-radiative rate).

Since, we are interested in the time required by the molecule to return to the ground state from the excited state, we can start with the initial condition that, the molecules are in excited state *S*_1_. Note that, the time required for excitation is orders of magnitude smaller than the emission timescale. Accordingly, one can drop the first term (*f_ill_A N*_*S*0_(*t*)) on the right-hand side of first rate equation. The resultant rate equation for the excited state population *N*_*S*1_ becomes,

(2) $$\frac{d}{dt}N_{S1}(t)=-(k_{f}+k_{nr})\;N_{S1}(t)$$

Integration and simplification gives,

(3) $$\begin{array}{l} \int \frac{dN_{S1}(t)}{N_{S1}(t)}=-\int \left(k_{f}+k_{nr}\right)\;dt\\ log_{e}(N_{S1}(t))\;=-(k_{f}+k_{nr})t+C \end{array}$$

Now, we divide both the sides by log_10_ *e* to convert the solution in log_10_-scale,

(4) $$\begin{array}{l} log_{10}(N_{S1}(t))=-\log_{10}e\;(k_{f}+k_{nr})\;t+C_{1}\\ \;N_{S1}(t)=10^{-\log_{10}e\;(k_{f}+k_{nr})t}\times 10^{C_{1}} \end{array}$$

where, *C*_1_ = *C* log_10_*e*.

Imposing the boundary condition that, *N*_*S*1_(*t*) = σ ρ*_N_ V_psf_* at *t* = 0, we get, *C*_1_ = log_10_ (σ ρ*_N_ V_psf_*). Substituting this in (3) gives,

(5) $$\begin{array}{l} \frac{N_{S1}(t)}{\left(\sigma \;\rho_{N}\;V_{psf}\right)}=10^{-\log_{10}e\;(k_{f}+k_{nr})\;t}\\ \;N_{S1}(t)=\sigma \;\rho_{N}\;V_{psf}\;10^{-t/\tau_{p}}. \end{array}$$

where,

(6) $$\tau_{p}=1/(k_{f}+k_{nr})\log_{10}e.$$

Since the output fluorescence intensity is proportional to the number of molecules in the excited state, one can rewrite the above equation in terms of output fluorescence,

(7) $$I(t)=I_{0}\;10^{-t/\tau_{p}}.$$

where, *I*_0_ corresponds to the intensity due to σ ρ*_N_ V_psf_* number of molecules in the excitation volume. It may be noted that, the above derivation is similar to the derivation for determining lifetime of fluorescent molecules.

Now, we investigate Equation (5) for two special cases:

**Case I:** For *t* = τ_*p*_, we get, *N*(*t* = τ_*p*_) = (σ ρ*_N_ V_psf_*) /10 and hence the corresponding intensity in ideal condition is, *I*(*t* = τ_*p*_) = *I*_0_ /10. This indicates the fact that, σ ρ*_N_ V_psf_*(1 − 1/10) = 0.9σ ρ*_N_ V_psf_* fraction of molecules (i.e., 90% of total excited molecules) in the focal volume emit before *t* = τ_*p*_ and 10% emit after *t* = τ_*p*_.
**Case II:** For *t* = 3τ_*p*_, *N*(*t* = 3τ_*p*_) = σ ρ*_N_ V_psf_* /1000 giving, *I*(*t* = 3τ_*p*_) = *I*_0_ /1000. This indicates that, 99.9% of total excited molecules emit before *t* = 3τ_*p*_ and so only 0.1% emit after 3τ_*p*_.

It is to be noted that, the limit on temporal resolution is determined by the time required for nearly cent-percent (i.e., 99.9%) molecules to relax to the ground state. As an example, for near cent-percent i.e., 99.9% emission, the molecules require at least (*t*_99.9%_ = 3τ_*p*_) time for completing the cycle (*S*_0_ → *S*_1_ → *S*_0_), assuming all the emitted photons are detected. *t*_99.9%_ is also the minimum time that the ensemble of molecules requires to prepare itself for the next excitation-emission cycle. This can be considered as the temporal resolution limit and any detector/device that works faster than this time (*t*_99.9%_ = 3τ_*p*_) would not be able to do better. This restriction is solely due to the molecular excitation-emission cycle of the ensemble of molecules in the focal spot. It may be noted that, the derivation is simple and similar to the derivation for determining the lifetime of fluorescent molecules. A much more sophisticated estimate can be obtained by considering other factors such as, photobleaching and scattering.

As an example, consider fluorescein in 0.1 M NaOH aqueous solution at a temperature of 35°C. The radiative and non-radiative rate constant for fluorescein are, 24× 10^7^ s^−1^ and 4.3× 10^7^ s^−1^ respectively (Arik et al., 2005). So, the temporal resolution [time required for almost all molecules (99.9%) to relax to ground state] in ideal condition is, *t*_99.9%_ = 3τ_*p*_ = 24.5 ns. On the other hand, one can think of quantum dots that can have a fluorescence lifetime of as long as 500 ns (Berezin and Achilefu, 2010), equivalent to a low total emission rate of approximately, *k_f_* + *k_nr_* = 2 × 10^6^ s^−1^. This suggests that, the temporal resolution while imaging with quantum dots can be as large as, *t*_99.9%_ = 3.45 μs. Similarly one can determine the temporal resolution limit 3 τ_p_ (using Equation 6) when imaging with the desired dye, provided the radiative and non-radiative rate constants are known.

## 3. Imaging systems capable of high temporal resolution

### 3.1. Multifocal multiphoton microscopy

Multifocal microscopy is probably the first fluorescence imaging technique that brings in the concept of high temporal resolution. Multiphoton excitation takes place in the focal region where the high photon flux makes possible the simultaneous interaction of two photons with the target molecules. With the availability of powerful lasers (Ti-Sapphire Laser) of average power of ≈1 W/cm^2^ and peak power in few hundred Gigawatts, it becomes attractive to think of ways to accelerate the imaging process by splitting the beam so as to generate several focal spots. This facilitates large area parallel scanning of the specimen.

In ideal condition, the signal from the focus of a n-photon excitation (nPE) microscopy system is directly proportional to the *n*th order of average laser power *P_a_*, and inversely proportional to (*n*−1)th order of pulse width (τ) and repetition rate (*f*) i.e.,

(8) $$I_{nPE}=\sigma_{n}\;\frac{P_{a}^{n}}{{\left(\tau \;f\right)}^{n-1}}$$

where, σ_*n*_ is the n-photon absorption cross-section.

In case of MMM system, the laser beam is split into M-beams so the average power gets scaled by *M* i.e., the power for each beam in MMM system is, *P_MMM_* = *P_a_*/*M*. So the signal for overall MMM system of M-beams is,

(9) $$I_{MMM,\;nPE}=\sigma_{n}\;\frac{P_{MMM}^{n}}{{\left(\tau \;f\;M\right)}^{n-1}}$$

where, σ_*n*_ is the n-photon cross-section.

Without going into unnecessary details, we focus our attention to 2-photon excitation. So, for an MMM system for which the excitation is based on 2-photon absorption, the output signal emerging from each focused region is given by,

(10) $$I_{MMM,\;2PE}=\sigma_{2}\;\frac{P_{MMM}^{2}}{\tau \;f\;M}$$

where, σ_2_ is the 2-photon absorption cross-section.

For a typical MMM-system, the degree of parallelization is decided by the ratio of average power available and the saturation at the focus i.e., *N*_parallel_ = *P_a_* /*P_s_*. Here, *P_s_* is the maximum available saturation level at the focus.

**Figure 1:**
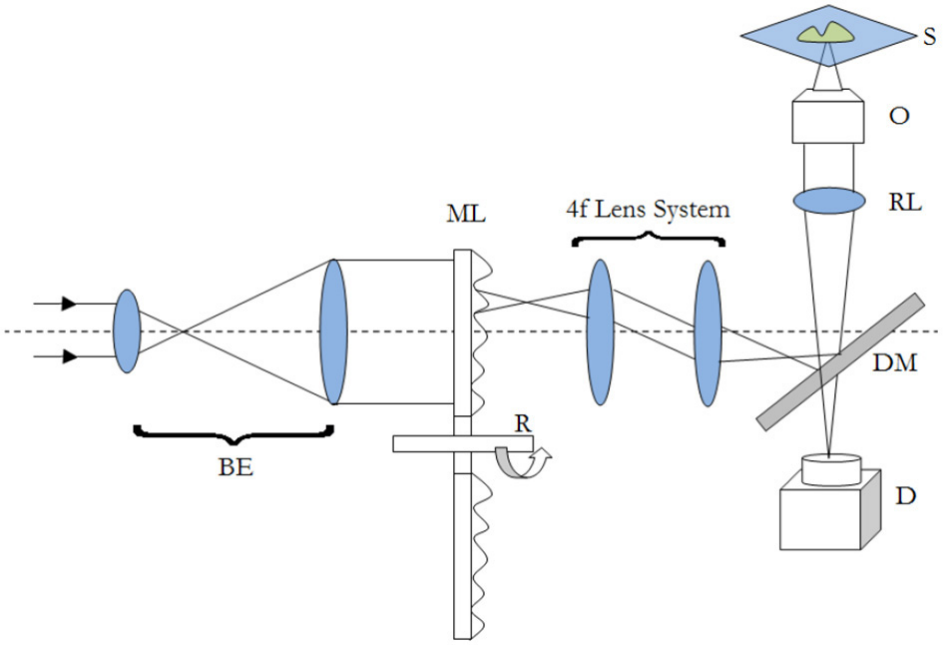
**Schematic diagram of a typical MMM imaging system**. The abbreviations are defined as: BE, Beam-Expander; ML, Micro-Lens; R, Rotating Disk; DM, Dichroic Mirror; RL, Relay Lens; O, Objective; S, Specimen; D, Detector.

**Figure 2:**
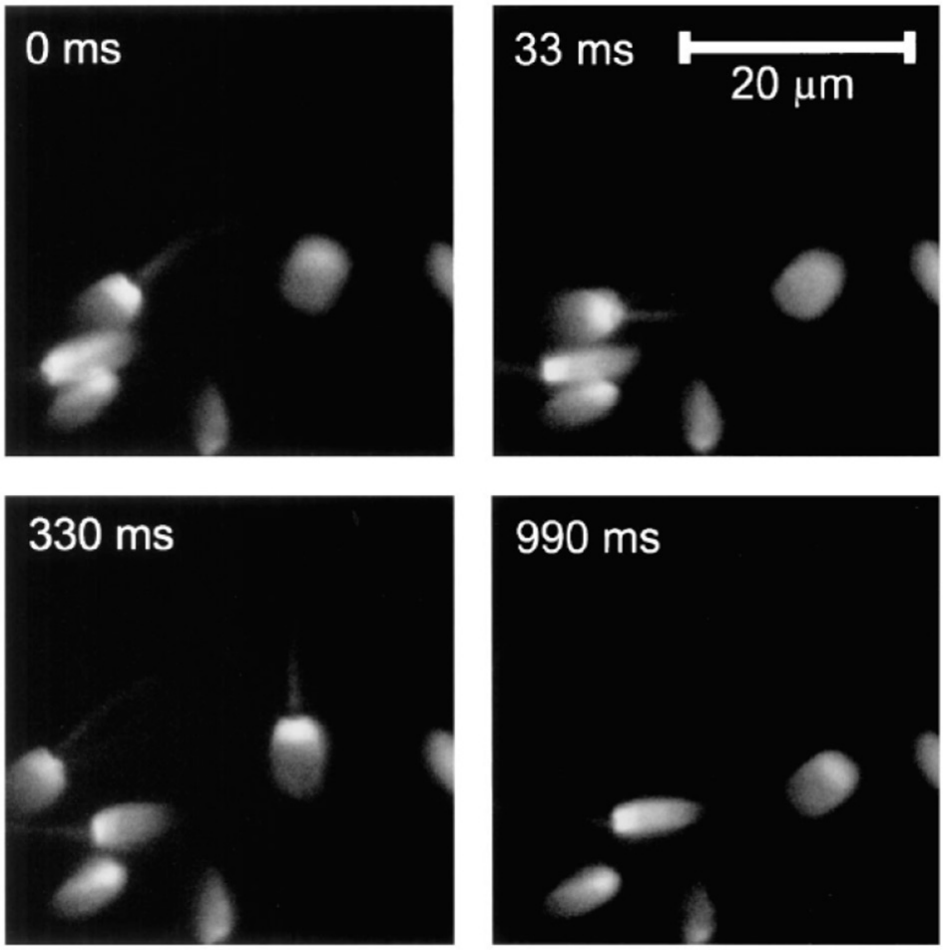
**2D images of live boar-sperm cells taken within 33 ms (Bewersdorf et al., 1998)**. Four typical images at different timescales (0, 33, 330, 990 ms) of rapidly moving cells are shown.

**Figure 3:**
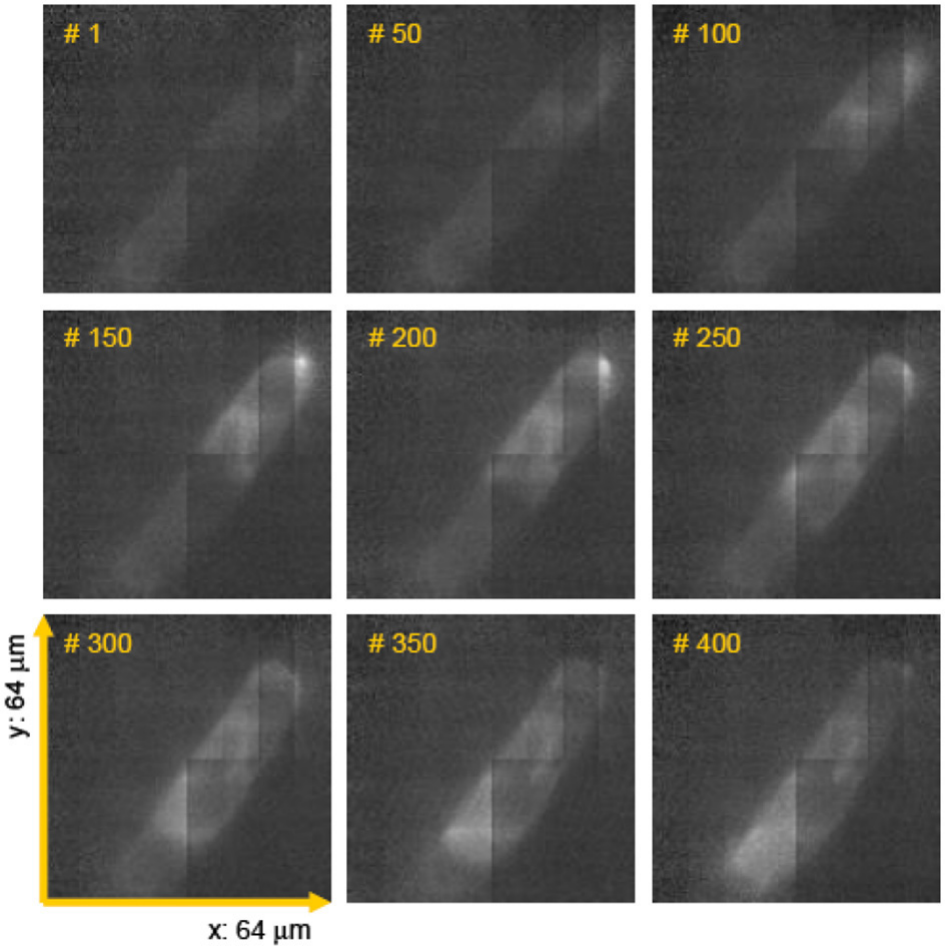
**Few frames of a movie (consists of 400 images taken in 0.625 s) showing spontaneous contraction of cardiac myocytes (labeled with fluo3 dye) (Bahlmann et al., 2007)**.

One of the typical realization of MMM-system is schematically shown in Figure 1. There are two parts: first, the ML array and second, the high resolution inverted fluorescence microscope. The laser beam is appropriately expanded using a beam-expander (BE) to fill the ML array. This results in the generation of many tiny focal spots which are directed to the dichoric mirror (DM) using a 4f lens system. The rays subsequently reach the back-aperture of the objective lens through relay lens (RL). For simplicity, ray diagram for only one of the micro-lens is shown in Figure 1. Similarly, an array of discrete focus points are created in the focal plane by other micro-lenses. Subsequently, the fluorescence from each of the points are collected by the same objective lens (O) and focused to the detector (D). The dichroic mirror filters out the incident light and allows the Stoke-shifted fluorescence light to pass-through. This system results in simultaneously imaging of many points on the image-plane in a single scan. The disk can be rotated (see Figure 1) to collect the complete data. In this way fast data-collection can be achieved. As an example, Figure 2 show xy-images of live boar-sperm cells that are obtained within 30 ms using MMM system (Bewersdorf et al., 1998). The head and tail of the cells are respectively labeled with Hoechst 33342 and fluorescein. A stack of 191 images were reportedly obtained within 6 s to monitor for the movements of the cells in the aqueous medium (Bewersdorf et al., 1998). A recent version of MMM system indicates a frame rate of >600 Hz (Bahlmann et al., 2007). Using this system, contraction of cardiac myocytes (labeled with Fluo3 dye) were obtained at an incredible fast rate of 640 Hz. Figure 3 show the image acquisition with a time of 1.56 ms. 400 images of size 64 × 64 μm were respectively taken in 0.625 s (Bahlmann et al., 2007).

It may however be noted that, there are many more realizations of MMM system (Pawley, 2006). Very recent developments have been, the realization of MMM system using spatial light modulator (Shao et al., 2012). Advanced versions of MMM system include, fluorescence lifetime imaging and second harmonic generation that are capable of generation multi-focal lifetime and second-harmonic images much faster than the classical techniques. While MMM system have many advantages they have limitations too. The most prominent one is the crosstalk arising from the overlap of the fields from multiple foci that result in optical aberration. Another limitation is the inability to obtain reliable image of scattering specimen.

### 3.2. Multifocal plane microscopy

**Figure 4:**
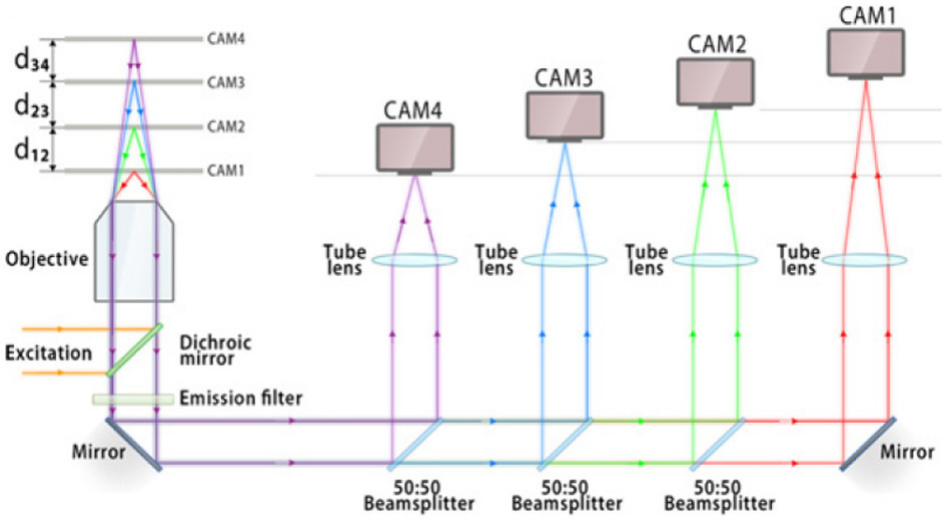
**Schematic diagram showing the optical layout of multifocal plane microscopy and illustrating the working principle (Ram et al., 2012)**. The fluorescence signal is focused onto an imaging detector positioned at a specific calibrated distance from the tube lens, thereby allowing simultaneous imaging of multiple focal planes. This enables simultaneous visualization and tracking of rapidly occuring cellular events.

To be able to simultaneously visualize multiple specimen planes is of utmost importance in microscopy and imaging. Multifocal plane microscopy achieves this in a very simplistic way (Ram et al., 2012; Prabhat et al., 2004). A schematic diagram of this technique is shown in Figure 4. The sample is illuminated by a laser of appropriate wavelength. The light is reflected by the dichoric mirror at the back-aperture of the objective lens. The emitted fluorescence from the specimen passes through the a set of filters to get rid of scattered light. The light is then equally split into four 50:50 beams using a series of beam-splitters. At a specific calibrated distance, the fluorescence light is focused onto a CCD/CMOS detector. This enables simultaneous visualization of 4 specimen planes. Experimentally, the axial spacing *d*_12_, *d*_23_, *d*_34_ reported between the focal planes are, 1.3, 1.6, and 1.55 μm respectively (Ram et al., 2012).

**Figure 5:**
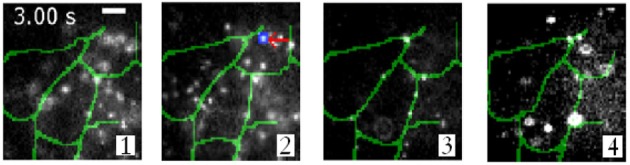
**Intercellular transfer of Tf molecules and its tracking in Z310 cell monolayer (Ram et al., 2012)**. Overlay of Tf-QD channel (gray scale) acquired from a live-cell monolayer using a 4-plane setup and the segmented plasma membrane channel (green). Each image corresponds to a distinct focal plane within the monolayer.

Using this imaging modality, Ram et al. show the intercellular transfer of transferrin molecules in Z310 cell monolayer as shown in Figure 5 (Ram et al., 2012). The four images correspond to images obtained form distinct focal plane within the monolayers. The quantum dot labeled single Tf-molecule is seen endocytosed by the adjacent recipient cell in the monolayer. This gives a deep insight for long-range intercellular transport observed for this molecule (Ram et al., 2012).

### 3.3. Multiplane microscopy

Another simple yet efficient technique to simultaneously visualize images from different specimen planes is multiplane microscopy (Dalgarno et al., 2010). A combination of distorted diffraction grating and lens placed in the detection sub-system. Distortion of lines in a grating allows order-dependent focusing power. This means that, negative focusing power is produced in the negative diffraction order, positive focusing power in the positive order and leaves the zeroth-order unchanged. It may be noted that, this is very different from the normally available diffraction grating that has uniform parallel grating and results in order-dependent change of direction to a light beam. Effectively, such a specialized quadratic grating can image multiple z-planes onto a single image plane of the detector (CCD/CMOS).

**Figure 6:**
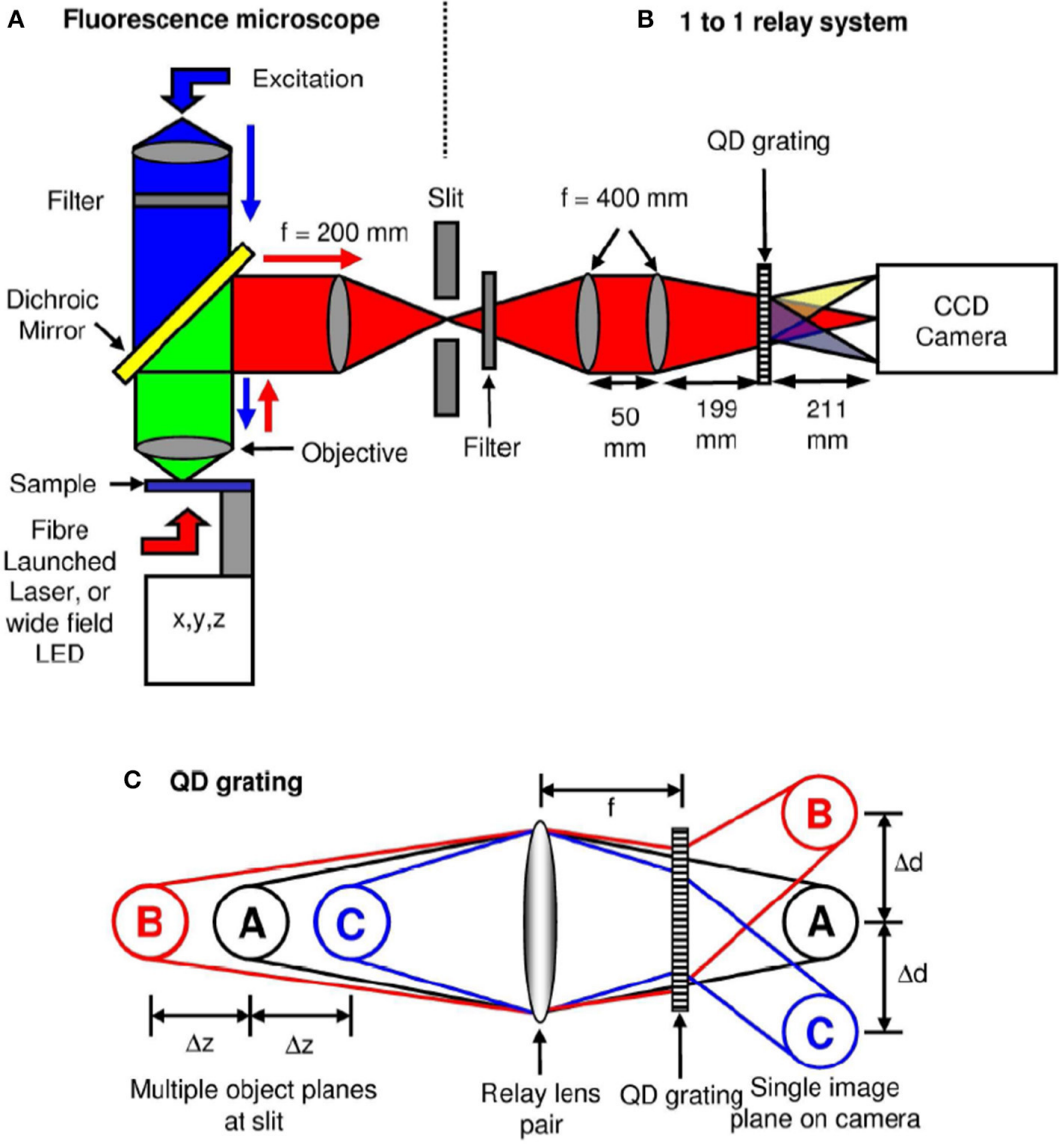
**Schematic diagram of the optical microscope and grating operation (Dalgarno et al., 2010). (A)** A typical wide-field 100× fluorescence microscopy system. **(B)** A relay system along with quadratically distorted grating, **(C)** Schematic diagram showing simultaneously imaging multi-layer images onto a single image-plane.

**Figure 7:**
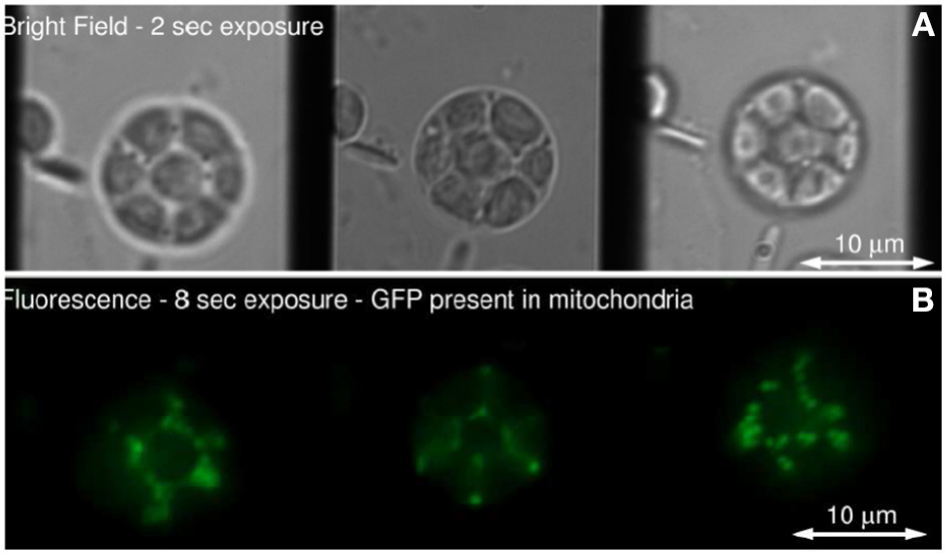
**(A)** Single Arabidopsis protoplast imaged in bright field, **(B)** Fluorescence from mitochondrial-targeted GFP (Dalgarno et al., 2010).

Figure 6 show the schematic of such a system integrated with inverted fluorescence microscope. The detection part is modified where a combination of focusing lens and qudratic distorted grating is inserted before the CCD camera. The grating splits the light emerging from different planes of the specimen onto the single plane camera. The separation between the object planes (range from few to few tens of microns) is determined by the magnification of the entire system, focal length of the relay optics and more importantly the properties of the quadratic grating. In the image plane (CCD camera), this produces side-by-side images corresponding to diffraction orders, *m* = −1, 0, +1 (see Figure 6C). Note that, the axial separation of the sample-planes is given by, Δ*z_m_* = *f*^2^_*R*_ /*M*^2^*_sys_f_m_*, where, *f_R_* and *f_m_* are the focal length of the relay optics and focal-power of the *m*th diffraction order; M is the magnification of the system (Dalgarno et al., 2010). Multiple plane imaging of single cell is demonstrated using this technique. Single plant cell Arabidopsis protoplast was imaged by expressing GFP that targeted mitocondria. Figure 7 show both widefield and fluorescent images obtained at different object planes, *z* = −0.9, 0.0, +0.9 microns. The specimen was illuminated with 510 nm light in the widefield mode whereas, fluorescent images were acquired with 470 nm laser light. Mitochondrial distribution at different focal depths is clearly visible in the images.

### 3.4. Multiple excitation-spot optical (MESO) microscopy

As the name suggests, multiple excitation spot optical microscopy is an imaging technique that enables simultaneous visualization of multiple specimen planes (Partha, 2009; Partha and Alberto, 2011). This feature makes it a unique tool for simultaneous monitoring and tracking many particles for understanding complex biological processes. Unlike other techniques, this has the inbuilt potential for enabling multi-particle tracking.

**Figure 8:**
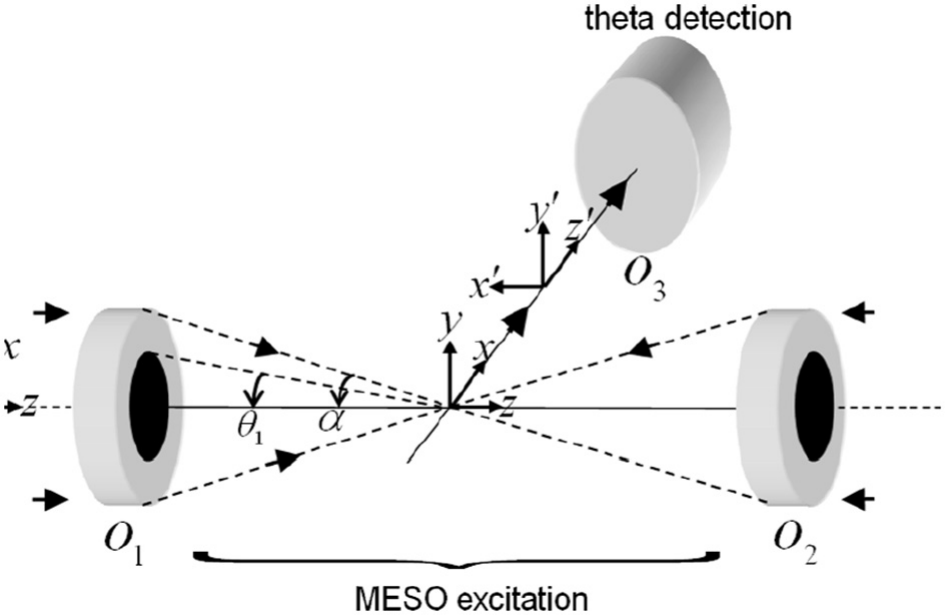
**Schematic diagram describing the optical configuration of MESO microscopy (Partha and Alberto, 2011)**.

The schematic diagram describing a MESO imaging system is shown in Figure 8. The illumination consists of objective lens *O*_1_ and *O*_2_ arranged in a 4π-geometry. A spatial filtering technique is employed in which both the objective lens are aperture engineered. The spatial filter is purposefully designed to produce an annular/ring illumination at the back of the objective lens. The objective lens approximately performs a Fourier transform, giving rise to Bessel-like beam when illuminated with a ring-illumination pattern. A similar counter-propagating phased-matched Bessel-like beam is generated by the aperture-engineered objective *O*_2_. The super-position of both beams at the focal volume gives rise to an interference pattern that represents a patterned array of nanodots. Orthogonal detection may be employed to detect fluorescence from individual nanodots. This can be achieved by placing a detection objective *O*_3_ at 90° to the illumination arm (see Figure 8). The detection object is moved along the optical z-axis to collect fluorescence from individual nanodots.

The illumination and detection PSF determines the overall system point spread function (PSF) of the MESO imaging system. Note that, the illumination is aperture-engineered in a 4π-geometry. The *x*-, *y*- and *z*- components of the electric field for randomly polarized light illumination in Cartesian coordinate system are given by Wolf and Richards (1959), Biovin and Wolf (1965), and Partha and Alberto (2011),

(11) $$\left[\begin{array}{l} E_{x}\\ E_{y}\\ E_{z} \end{array}\right]=\left[\begin{array}{l} -iA(I_{0}+I_{2}\cos(2\phi ))\\ -iAI_{2}\sin(2\phi )\\ -2AI_{1}\sin(\phi ) \end{array}\right]$$

where *A* is the proportionality constant representing the amplitude of the incident electric field.

For randomly-oriented dipoles, the excitation PSF of the MESO system is,

(12) $$\begin{array}{l} h_{ill}(x,y,z)=|\bar{E}(x,y,z)+\bar{E}(x,y,-z){|}^{2}\\ \;=|Re{\tilde{I}}_{0}{|}^{2}+2|Re{\tilde{I}}_{1}{|}^{2}+|Re{\tilde{I}}_{2}{|}^{2} \end{array}$$

where, the modified diffraction integral over the aperture angle α_*ill*_ are given by,

$${\tilde{I}}_{0,1,2}=\int_{\theta =0}^{\alpha_{ill}}B(*)G_{0,1,2}(*)\cos^{1/2}\theta \;e^{i\left(\frac{u\cos\theta }{\sin^{2}\alpha_{ill}}\right)}d\theta$$

and,

$$\left[\begin{array}{l} G_{0}(*)\\ G_{1}(*)\\ G_{2}(*) \end{array}\right]=\left[\begin{array}{l} \sin\theta (1+\cos\theta )J_{0}\left(\frac{v\sin\theta }{sin\alpha }\right)\\ \;\sin^{2}\theta J_{1}\left(\frac{v\sin\theta }{sin\alpha }\right)\\ \;\sin\theta (1-\cos\theta )J_{2}\left(\frac{v\sin\theta }{sin\alpha }\right) \end{array}\right]$$

The spatial filter is characterized by the optical mask with transmission function,

(13) $$B(*)=H[\theta -\theta_{1}]-H[\theta -\alpha ]$$

where, *H*(*) is the Heaviside function. The cutoff angle is, θ_1_, for which the transmission window is Δθ = 5°. $u=\frac{2\pi }{\lambda }z\sin^{2}\alpha$ and $v=\frac{2\pi }{\lambda }x^{2}+y^{2}{}^{1/2}\sin\alpha$ are the longitudinal and transverse optical coordinates, respectively (Wolf and Richards, 1959; Biovin and Wolf, 1965), and α_*ill*_ is the illumination semi-aperture angle.

The technique employs a theta detection scheme, in which the detection is carried out in the orthogonal plane, as shown in Figure 1. The orthogonal plane is represented by the following transformation: (*x*′ = −*z*, *y*′ = *y*, *z*′ = *x*). This detection scheme has the advantage of high resolution at long working distance. The isotropic emission model is assumed with randomly polarized light excitation. The components of the detection PSF are,

(14) $$h_{det}(x^{\prime },y^{\prime },z^{\prime })=|{\bar{E}}_{x^{\prime }}{|}^{2}+|{\bar{E}}_{y^{\prime }}{|}^{2}+|{\bar{E}}_{z^{\prime }}{|}^{2}$$

where the integration $I_{0,1,2}=\int_{\theta =0}^{\alpha_{det}}\left(\ldots \right)d\theta$ on the aperture-free objective (*O*_3_) is carried over the detection semi-aperture angle α*_det_*.

Overall, the system PSF of the proposed imaging system is given by,

(15) $$h_{sys}(x,y,z)=h_{ill}(x,y,z)\times h_{det}(-z,y,x).$$

**Figure 9:**
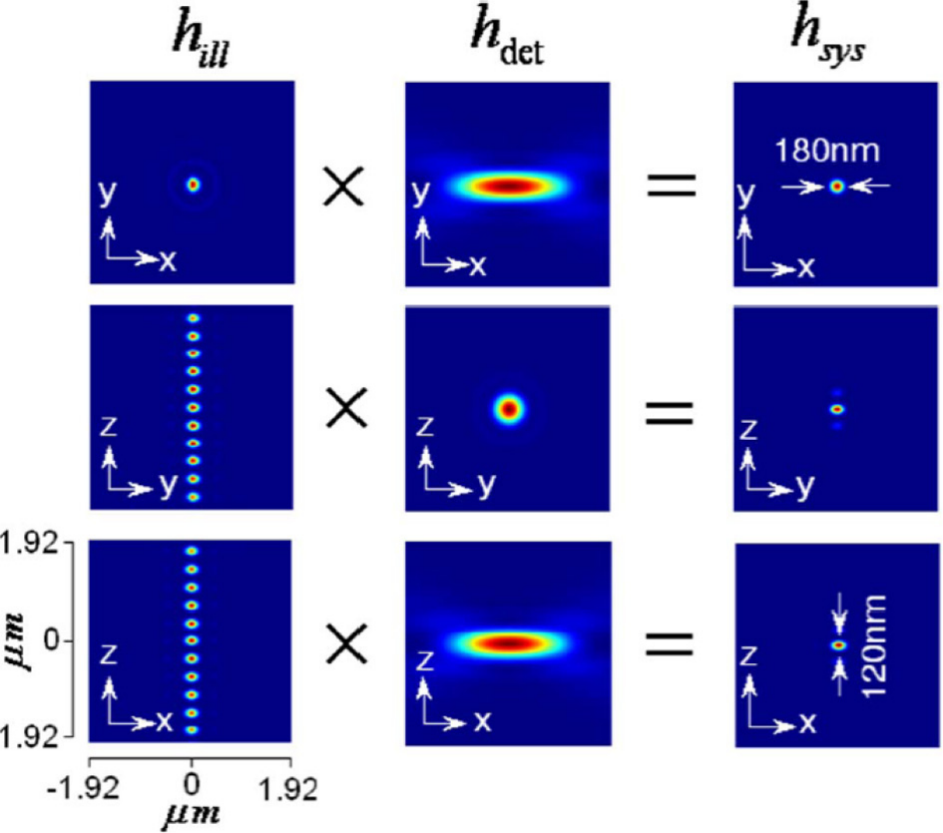
**Illumination, detection and system PSF for MESO system (Partha and Alberto, 2011)**.

**Figure 10:**
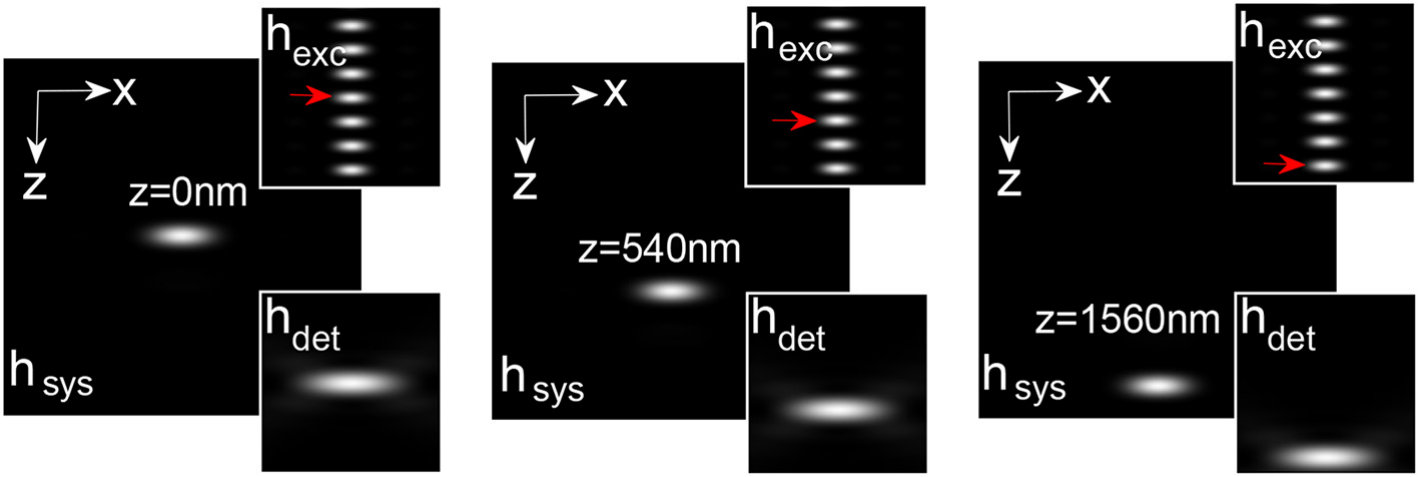
**Detection of nanodots at varying z-layers: *z* = 0, 540, and 1560 (Partha and Alberto, 2011)**.

The illumination, detection and system PSF for the proposed *MESO* system are shown in Figure 9 for α_*ill*_ = 45° and α*_det_* = 45°. One can immediately note that, the axial resolution of the proposed system is improved by a factor of approximately 4 over the classical resolution limit, while there is no improvement in the lateral resolution because the perpendicular detection occurs along the lateral axis of the illumination objective. Study show that, the axial and lateral resolutions are approximately 120 and 180 nm, respectively (Partha and Alberto, 2011). In principle, the fluorescence from all of the nanodots can be recorded by scanning the objective *O*_3_. It is important to obtain information from individual nano-dots at varying depths (z-axis). The detection of target nanodot situated at depths of, *z* = 0 nm, 540 nm, and 1.56 μm is shown in Figure 10. The red arrows indicate the target nanodot. In principle, simultaneous detection from all nanodots can be obtained by using appropriate optical elements, such as a distorted diffraction grating in the detection path of the imaging system (Dalgarno et al., 2010). Such a system has the capability for near-simultaneous in-depth multilayer imaging of biological specimens, thereby increasing the temporal resolution of the imaging system. For example, to scan *M* slices of an *N* × *N* image, a laser scanning system requires *M* × *N*^2^ scan-points and a total time of (*M* × *N*^2^)Δ*t*. MESO system with *M* axial dots, theoretically allows the data acquisition rate to be increased by a factor of *M*, thereby increasing the temporal resolution by a factor of *M* Δ*t*.

### 3.5. Multiple light sheet microscopy (MLSM)

This microscopy technique involves the generation of multiple light-sheets and imaging. A spatial filtering technique is employed in a selective plane illumination microscopy (SPIM) setup to generate multiple light sheets. The technique is robust and paves the way for volume imaging in fluorescence microscopy (Mohan et al., 2014).

**Figure 11:**
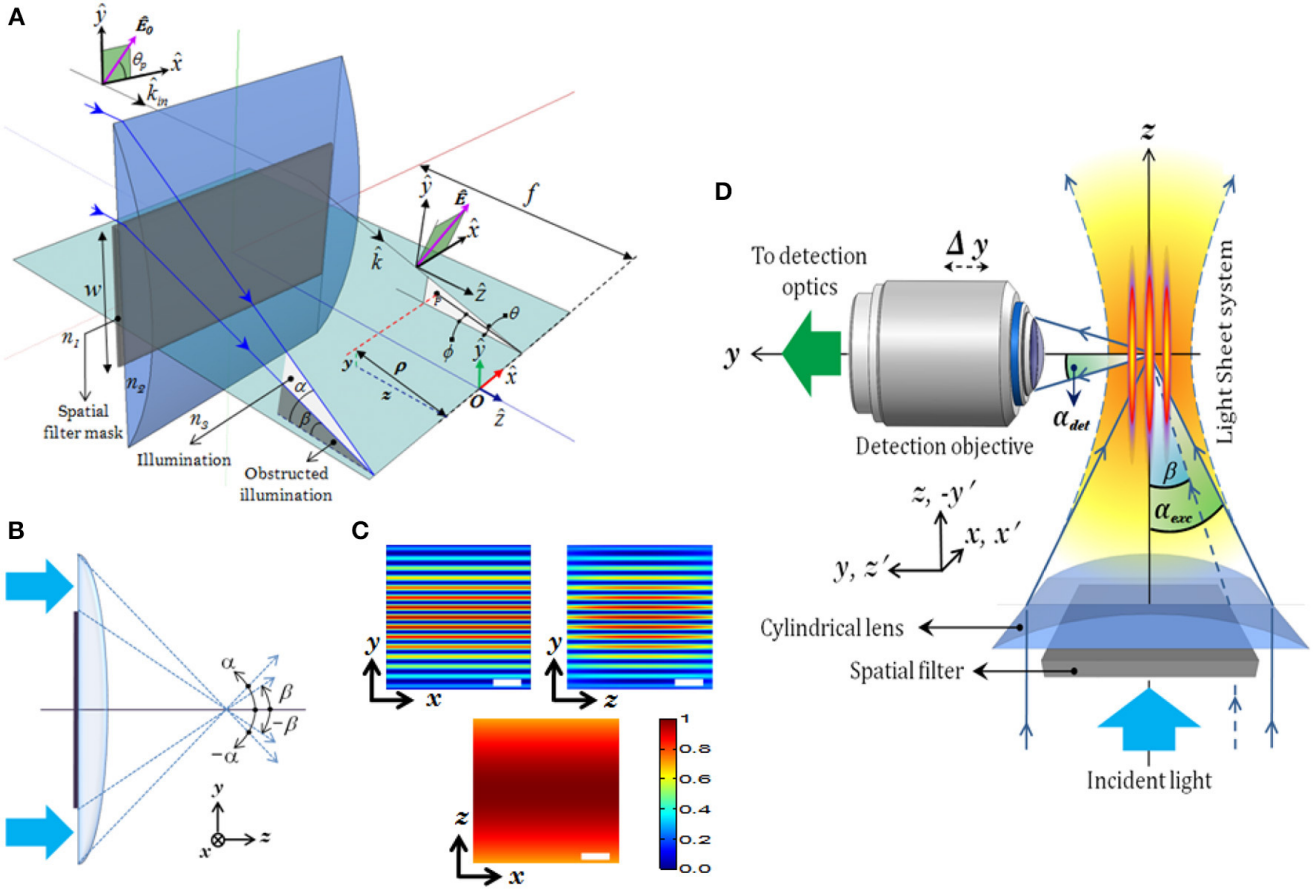
**(A)** Schematic diagram of the excitation part describing all the imaging parameters (Mohan et al., 2014). **(B)** Spatial filter. **(C)** *XY*, *YZ*, *XZ* planes of excitation PSF (imaging parameters: λ_exc_ = 532 nm, α_exc_ = 30°, β = 25°), **(D)** Schematic diagram of the complete imaging system with orthogonal detection. Scale-bar = 1.5 μm.

The system PSF is solely determined by the structure of the spatial filter employed in a cylindrical lens system. The incident plane wave is subjected to rectangular spatial filter that allows the light to pass through the periphery (see Figure 11A). The structured wavefront thus created was incident at the back aperture of the cylindrical lens. Since the cylindrical lens performs 1D Fourier transform, this results in multiple light-sheets at and off the focus. The light-sheets differ in intensity but can be tailored by adjusting the parameters of spatial filter. The resulting field distribution can be calculated at and near the focus. In cylindrical coordinates (ρ, ϕ, *z*) with primary axis of the cylindrical lens along *x*−direction, the electric field components at the focus for a linearly polarized light illumination (polarization angle θ_*p*_ with *x*-axis) with profile *E*_in_(θ) is given by Purnapatra and Mondal (2013),

(16) $$\left[\begin{array}{c} E_{x}(\rho ,\phi )\\ E_{y}(\rho ,\phi )\\ E_{z}(\rho ,\phi ) \end{array}\right]=A\int_{-\alpha }^{\alpha }E_{in}(\theta )\left[\begin{array}{c} \cos\theta_{p}\\ \sin\theta_{p}\cos\theta \\ \sin\theta_{p}\sin\theta \end{array}\right]\cos^{\frac{1}{2}}\theta e^{\left[i\rho k\{\cos(\theta -\phi )\}\right]}\text{d}\theta \text{​​}$$

where, α is the semi-aperture angle of the lens defined by it's numerical aperture, *k* is the wavenumber in the image space and $A=\sqrt{\frac{fk}{2\pi }}e^{-ifk}e^{i\pi /4}\sqrt{\frac{n_{1}}{n_{3}}}$. The terms *f*,*n*_1_ and *n*_3_ denote the focal length, refractive index of object and image space respectively while the radial distance from the *x*−axis and the polar inclination are denoted by, $\rho =\sqrt{y^{2}+z^{2}}$ and ϕ = tan^−1^(*y*/*z*) respectively. By introducing an amplitude transmission function *T*(θ), |θ| ≤ α (that of spatial filter), Equation (16) modifies to,

(17) $$\left[\begin{array}{c} E_{x}\\ E_{y}\\ E_{z} \end{array}\right]=A\int_{-\alpha }^{\alpha }E_{in}(\theta )T(\theta )\left[\begin{array}{c} \cos\theta_{p}\\ \sin\theta_{p}\cos\theta \\ \sin\theta_{p}\sin\theta \end{array}\right]\sqrt{\cos\theta }e^{\left[i\rho k\{\cos(\theta -\phi )\}\right]}\text{d}\theta \text{​​​​}$$

The corresponding transmission function *T*(θ) of the spatial filter that is suitable for generating multiple light-sheets is given by,

(18) $$T(\theta )=\{\begin{array}{ll} 1, & \text{if }\beta <|\theta |\leq \alpha \\ 0, & \text{if }|\theta |\leq \beta \end{array}$$

The schematic diagram of MLSM microcopy system is shown in Figure 11D. The system can be broadly split into two independent optical configurations: excitation sub-system and detection sub-system. An orthogonal detection system is employed which embodies many advantages over the existing detection techniques. Light of wavelength λ_exc_ = 532 nm is allowed to pass through the spatial filter [transmission function (*T*_θ_)] to generate a structured wavefront, *S*(θ) = *A*(θ)*T*(θ), where, *A*(θ) = 1 for plane wave. The excitation sub-system and the spatial filter along with the imaging parameters, are shown in Figures 11A,B respectively. The illumination PSF of the proposed imaging modality is obtained using Equation (16) and the result is shown in Figure 11C. A central light-sheet, along with one on either side, is clearly evident. Although weak, the intensity of off-focus light-sheets can be boosted by tuning the stop angle (β) of the spatial filter. This facilitates simultaneous excitation of multiple specimen layers. The intensities of the central and a few nearby sidelobes are fairly high, but fall off gradually with distance from the central light sheet. This can be explained based on the 1D Fourier transform (performed by the cylindrical lens) of a rectangular window function (spatial filter), which is a *S*inc function (intensity distribution at focus). This further evidences the fact that, one can reliably scan 5–7 layers of the specimen simultaneously. This is a step closer to volume imaging. The complete MLSM system that comprises both illumination and detection sub-systems is shown in Figure 11D. The fluorescence light emanating from the specimen is collected by the detection objective placed orthogonal to the illumination arm. The light is subsequently filtered to remove the scattered light and focused on the CCD camera.

**Figure 12:**
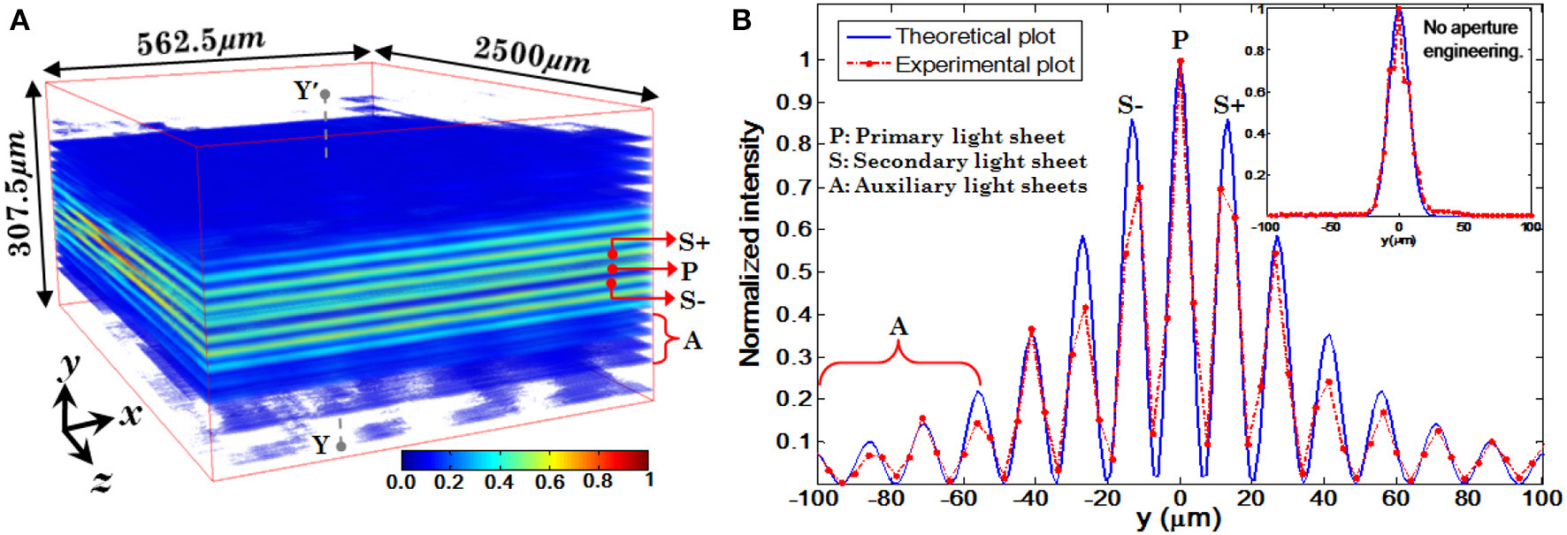
**(A)** Volumetric rendition of the experimentally obtained PSF (spatial filter parameters: λ_exe_ = 532 nm, α = 1.17°, β = 0.99°, θ_*p*_ = 0°). **(B)** Intensity plots of experimentally obtained illumination PSF and its comparison with simulated PSF (Purnapatra et al., 2014).

**Figure 13:**
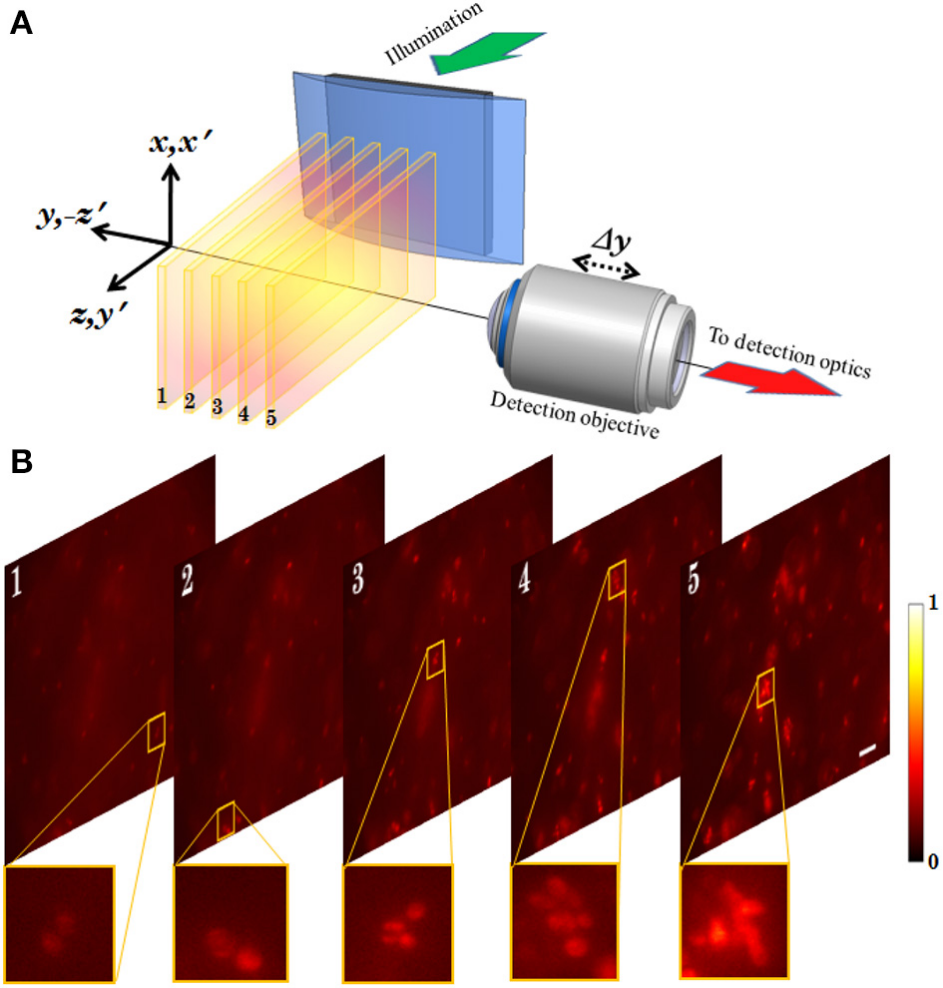
**(A)** Schematic diagram showing the optical setup of the MLSM system (Mohan et al., 2014). **(B)** 2D images obtained from different planes of the specimen probed by multiple light-sheet pattern. Scalebar: 25 μm.

Experimentally measured 3D structure of the optical field of the multi-layer illumination system is shown in Figure 12A (Purnapatra et al., 2014). One can observe the central light-sheet and its immediately neighboring light-sheets (denoted by *P* and *S*_+_, *S*_−_) along with the distant light-sheets (denoted by *A*) along the slice direction (y-axis). Intensity plots are shown in Figure 12B (red broken lines). For comparison, corresponding theoretically calculated plots are also shown (by blue lines). It must be noted that this plot has been corrected for the fact that the laser intensity profile is Gaussian. The SPIM system with the same parameters is shown in the inset of Figure 12B. We used MLSM and a dedicated orthogonal detection system to realize the imaging system (Mohan et al., 2014). This is accomplished by translating the detection arm in order to focus on a specific light-sheet in a selective manner. The results on an agarose gel sample with suspended yeast cells (coated with NHS-Rhodamine tagged polymer) are shown in Figure 13 (Mohan et al., 2014) Translation along +*z*′ axis revealed a series of in-focus and out-of-focus image planes. The in-focus planes are observed as a result of intersection of a particular light-sheet with the detection PSF. Note that, we also observed out-of–focus fluorescence from other specimen planes. The background is created by other light-sheets separated by some distance along the *y*− axis which appear as defocused objects. A detection system along with high NA objective (that shrinks the detection PSF) can be employed to reduce the contribution from out-of-focus light. Overall the proposed system may enable simultaneous monitoring of different organ growth during the developmental process and expedite volume imaging.

## 4. Conclusions

In this feature article, I derived an approximate expression to determine the temporal resolution of a 3-level molecular system (dye molecule). When imaging with a characteristic dye molecule in fluorescence microscopy, we determined that the temporal resolution depends on the emission-excitation cycle of the fluorophore. The temporal resolution is essentially determined by the time taken for 99.9% of the excited molecules to return back to ground state. Further, I discussed some of the prominent optical microscopy systems that are capable of visualizing multiple specimen layers, thereby improving the temporal resolution by many-folds. Comparison of these microscopy techniques are essential to understand the advantages and disadvantages. Micro-lens array based MMM system and its variants are capable of very large frame rates (Bahlmann et al., 2007). But this technique is not able to simultaneously image multiple specimen layers. On the other hand, multifocal plane microscopy enables simultaneous visualization of 4 specimen planes (Ram et al., 2012). A similar technique termed as, multiplane microscopy can image multiple planes (3 planes) at varying depths with a inter-plane spacing of approximately 1 micron (Dalgarno et al., 2010). Interference based technique such as, MESO microscopy is capable of simultaneous imaging >10 specimen planes (Partha and Alberto, 2011). A two photon version of this technique has the added advantage of negligible cross-talks. A relatively new technique is multiple light-sheet microscopy (MLSM) which is primarily based on light-sheet illumination and orthogonal detection (Mohan et al., 2014). This technique is capable of capturing the whole volume simultaneously. Experimentally, imaging of 5 specimen layers has been achieved using this technique. We assert that such, such systems can give rise to rapid volume imaging with minimal photobleaching and photodamage.

### Conflict of interest statement

The author declares that the research was conducted in the absence of any commercial or financial relationships that could be construed as a potential conflict of interest.

## References

[B1] Abbe E. (1873). Beitrge zur Theorie des Mikroskops und der mikroskopischen Wahrnehmung. Arch. f.Mikr. Anat. 9, 413–420 10.1007/BF02956173

[B2] Arik M. Celebi N. Onganer Y. (2005). Fluorescence quanching of Fluorescein with molecular oxygen in solution. J. Photochen. Photobiol. A 170, 105–111 10.1016/j.jphotochem.2004.07.004

[B3] Bahlmann K. So P. T. Kirber M. Reich R. Kosicki B. McGonagle W.. (2007). Multifocal multiphoton microscopy (MMM) at a frame rate beyond 600 Hz.İ Opt. Exp. 15, 10991–10998. 10.1364/OE.15.010991 19547456

[B4] Berezin M. Y. Achilefu S. (2010). Fluorescence lifetime measurements and biological imaging. Chem. Rev. 110, 2641–2684. 10.1021/cr900343z 20356094 PMC2924670

[B5] Betzig E. Patterson G. H. Sougrat R. Lindwasser O. W. Olenych S. Bonifacino J. S.. (2006). Imaging intracellular fluorescent proteins at nanometer resolution. Science 313, 1642. 10.1126/science.1127344 16902090

[B6] Bewersdorf J. Pick R. Hell S. W. (1998). Multiphoton multifocal microscopy. Opt. Lett. 23, 655–657. 10.1364/OL.23.000655 18087301

[B7] Biovin A. Wolf E. (1965). Electromagnetic field in the neighborhood of the focus. Phys. Rev. 138, B1561 10.1103/PhysRev.138.B1561

[B8] Dalgarno P. A. Dalgarno H. I. C. Putoud A. Lambert R. Paterson L. Logan D. C.. (2010). Greenaway, Multiplane imaging and three dimensional nanoscale particle tracking in biological microscopy. Opt. Exp. 18, 877–884. 10.1364/OE.18.000877 20173908

[B9] Deschout H. Zanacchi F. C. Mlodzianoski M. Diaspro A. Bewersdorf J. Hess S. T.. (2014). Precisely and accurately localizing single emitters in fluorescence microscopy. Nat. Methods 11:253. 10.1038/nmeth.2843 24577276

[B10] Fölling J. Bossi M. Bock H. Medda R. Wurm C. A. Hein B.. (2008). Fluorescence nanoscopy by ground-state depletion and single-molecule return. Nat. Methods 5, 943. 10.1038/nmeth.1257 18794861

[B11] Gustafsson M. G. L. (2005). Nonlinear structured-illumination microscopy: wide-field fluorescence imaging with theoretically unlimited resolution. Proc. Natl. Acad. Sci. U.S.A. 102, 13081. 10.1073/pnas.0406877102 16141335 PMC1201569

[B12] Hell S. W. Lindek S. Cremer C. Stelzer E. H. K. (1994). Measurement of the 4Pi-confocal point spread function proves 75 nm axial resolution. Appl. Phys. Lett. 64, 1335–1338 10.1063/1.111926

[B13] Hell S. W. Wichmann J. (1994). Breaking the diffraction resolution limit by stimulated emission: stimulated-emission-depletion fluorescence microscopy. Opt. Lett. 19, 780–782. 10.1364/OL.19.000780 19844443

[B14] Hess S. T. Girirajan T. P. K. Mason M. D. (2006). Ultra-high resolution imaging by fluorescence photoactivation localization microscopy. Biophys. J. 91, 4258. 10.1529/biophysj.106.091116 16980368 PMC1635685

[B15] Mohan K. Purnapatra S. B. Mondal P. P. (2014). Three dimensional fluorescence imaging using multiple light-sheet microscopy. PLoS ONE 9:e96551. 10.1371/journal.pone.0096551 24911061 PMC4050046

[B16] Partha P. M. (2009). Multi-focal multiphoton excitation fluorescence microscopy. Rev. Sci. Instrum. 80:096104 10.1063/1.3226658 19791975

[B17] Partha P. M. Alberto D. (2011). Simultaneous multilayer scanning and detection for multiphoton fluorescence microscopy. Nat. Sci. Rep. 1, 149. 10.1038/srep00149 22355665 PMC3240976

[B18] Pawley J. (2006). Handbook of Biologicl Confocal Microscopy, 3rd Edn. New York, NY: Springer 10.1007/978-0-387-45524-2

[B19] Prabhat P. Ram S. Ward E. S. Ober R. J. (2004). Simultaneous imaging of different focal planes in fluorescence microscopy for the study of cellular dynamics in three dimensions. IEEE Trans. Nanobiosci. 3, 237–242. 10.1109/TNB.2004.837899 15631134 PMC2761735

[B20] Purnapatra S. B. Mohan K. Mondal P. P. (2014). Generation of multiple sheets of light using spatial filtering technique. Opt. Lett. 39, 4715–4718. 10.1364/OL.39.004715 25121856

[B21] Purnapatra S. B. Mondal P. P. (2013). Determination of electric field at and near the focus of a cylindrical lens for applications in fluorescence microscopy. AIP Adv. 3:052124 10.1063/1.4807670

[B22] Ram S. Kim D. Ober R. J. Ward E. S. (2012). 3D single molecule tracking with multifocal plane microscopy reveals rapid intercellular transferrin transport at epithelial cell barriers. Biophys. J. 103, 1594–1603. 10.1016/j.bpj.2012.08.054 23062352 PMC3471454

[B23] Rust M. J. Bates M. Zhuang X. (2006). Sub-diffraction-limit imaging by stochastic optical reconstruction microscopy (STORM). Nat. Methods 3, 793. 10.1038/nmeth929 16896339 PMC2700296

[B24] Shao Y. Qin W. Liu H. Qu J. Peng X. Niu H.. (2012). Multifocal multiphoton microscopy based on a spatial light modulator. Appl. Phys. B 107, 653–657. 10.1007/s00340-012-5027-4 23894222 PMC3722068

[B25] Wolf E. Richards B. (1959). Electromagnetic diffraction in opti cal systems II. Structure of the image field in an aplanatic system. Proc. R. Soc. A 253, 358–379 10.1098/rspa.1959.0199

[B26] Zanacchi F. C. Lavagnino Z. Donnorso M. P. Del Bue A. Furia L. Faretta L. M.. (2011). Live-cell 3D super-resolution imaging in thick biological samples. Nat. Methods 8, 1047. 10.1038/nmeth.1744 21983925

